# A novel CXCL8(3–72) K11R/G31P–BPI_233_ hybrid protein modulates neutrophil-driven inflammation and protects against carbapenem-resistant *Acinetobacter baumannii* infection

**DOI:** 10.1080/22221751.2026.2741787

**Published:** 2026-10-15

**Authors:** Danni Pu, Wei Cao, Jindong Chen, Rongrong Song, Jiankang Zhao, Bin Cao

**Affiliations:** a China-Japan Friendship Hospital (Institute of Clinical Medical Sciences), Chinese Academy of Medical Sciences & Peking Union Medical College; b National Center for Respiratory Medicine, Beijing, People’s Republic of China; c State Key Laboratory of Respiratory Health and Multimorbidity, Beijing, People’s Republic of China; d National Clinical Research Center for Respiratory Diseases, Beijing, People’s Republic of China; e New Cornerstone Science Laboratory, Beijing, People’s Republic of China; f Institute of Respiratory Medicine, Chinese Academy of Medical Sciences, Beijing, People’s Republic of China; g Department of Pulmonary and Critical Care Medicine, Center of Respiratory Medicine, China–Japan Friendship Hospital, Beijing, People’s Republic of China; h Anjun Genetech Co., Ltd, Beijing, People’s Republic of China; i Changping Laboratory, Chinese Academy of Medical Sciences & Peking Union Medical College

**Keywords:** Carbapenem-resistant *Acinetobacter baumannii*, G31P–BPI_233_, neutrophils, endotoxin, pneumonia

## Abstract

Carbapenem-resistant Gram-negative bacteria (CRGNB) pose a severe global health threat. Carbapenem-resistant *Acinetobacter baumannii* (CRAB) is one of the most critical threats among CRGNB, and effective non-antibiotic therapeutic strategies are urgently needed. Bactericidal/permeability-increasing protein (BPI)-derived molecules display antibacterial and endotoxin-neutralizing activities against Gram-negative bacteria, whereas CXCL8(3–72) K11R/G31P (G31P), an analog of CXCL8 with the mutations of CXCL8 at K11 and G31 positions, could antagonize ELR-CXC chemokine-mediated neutrophil chemotaxis. Here, we engineered a dual-function fusion protein, G31P–BPI_233_, to combine BPI-mediated antibacterial activity with modulation of neutrophil-driven inflammation. Recombinant G31P–BPI_233_ was expressed in CHO-K1 cells and characterized through safety evaluation, endotoxin neutralization, bactericidal, serum killing, neutrophil killing, chemotaxis, cytokine, phagocytosis, and NET-associated marker assays, followed by validation in mouse models of CRAB septicemia and pneumonia. Short-term safety assessment suggested preliminary tolerability over 24 h, while G31P–BPI_233_ reduced residual endotoxin activity and exhibited concentration-dependent bactericidal activity against multiple Gram-negative bacteria. At molar-matched doses, it retained G31P-mediated inhibition of neutrophil migration and BPI_233_-associated bactericidal and cytokine-suppressive activities. In CRAB models, G31P–BPI_233_ improved septicemia survival and reduced pneumonia-associated bacterial burden and inflammatory injury, with endpoint-specific advantages over G31P, BPI_233_, or their equimolar mixture. Several pneumonia outcomes did not differ significantly between G31P–BPI_233_ and colistin. Mechanistic studies using BPI-deficient mice further demonstrated that endogenous BPI contributes to bacterial clearance, endotoxin control, and neutrophil maturation and effector function during CRAB infection. Collectively, these findings identify G31P–BPI_233_ as a promising host-directed antibacterial fusion protein for severe CRAB infection, with potential translational implications for the broader management of CRGNB.

## Introduction

Due to the inappropriate use of antibiotics, antibiotic resistance poses a significant threat to human health globally [1]. Among them, carbapenem-resistant Gram-negative bacteria (CRGNB) pose a major global threat to hospital-acquired infections, particularly among critically ill and immunocompromised patients [2]. These pathogens frequently cause hospital-acquired pneumonia, ventilator-associated pneumonia, and bloodstream infection [3]. Among CRGNB, carbapenem-resistant *Acinetobacter baumannii* (CRAB) is of particular concern because of its remarkable capacity to acquire antimicrobial resistance determinants, persist in hospital environments, and tolerate host- and treatment-associated stress [4,5]. The majority of *A. baumannii* isolates causing HAP/VAP globally are multidrug-resistant, with a pooled MDR prevalence of 79.9% (95% CI 73.9%–85.4%) [6]. Carbapenem resistance rates range from 30% to 90% in Asia, Eastern Europe, and Latin America, and in certain regions exceed 90%, with HAP and bloodstream infections due to CRAB resulting in mortality rates of up to 60% [7,8]. As the development of new antibiotics continues to lag behind the emergence of resistance, the development of novel non-antibiotic biological agents to address this unmet clinical need is particularly urgent [9].

Antimicrobial peptides represent promising therapeutic molecules, as they exhibit structural nuances and distinct molecular targets against pathogenic microorganisms [10]. Bactericidal/permeability-increasing protein (BPI) is a cationic antimicrobial protein stored mainly in neutrophil granules. It plays a crucial role in innate immunity against Gram-negative bacteria, with potent lipopolysaccharide (LPS)- binding and bactericidal activity [11–13]. The N-terminal region of BPI binds LPS, neutralizes endotoxin activity, and exerts direct bactericidal effects by damaging the bacterial outer membrane [14]. BPI-derived fragments and engineered BPI-based proteins have therefore been explored as anti-endotoxin and antibacterial agents. The recombinant N-terminal fragment of human BPI (rBPI21) was shown to be highly effective against MDR Gram-negative bacteria [15]. The BPI21/LL-37 fusion protein also enhanced bacterial clearance and LPS neutralization [16]. Although rBPI21 showed promising endotoxin-neutralizing and antibacterial properties, its clinical development was limited by high cost, a short half-life, and the difficulty of translating its sole endotoxin-neutralization effect into a robust survival benefit in heterogeneous septic patients [17,18]. Furthermore, previous BPI-derived therapeutics primarily focused on antibacterial and endotoxin-neutralizing activities. However, it could not directly modulate chemokine-driven neutrophil recruitment and excessive inflammatory injury during severe Gram-negative bacterial pneumonia.

Neutrophils are essential innate immune cells that play a pivotal role in early defense against Gram-negative bacterial infections through phagocytosis, intracellular killing, and the release of antimicrobial molecules and soluble mediators [19,20]. During Gram-negative pneumonia, neutrophil recruitment is orchestrated by multiple mediators, including chemokines such as CXCL8, complement, and leukotriene B4 (LTB4) [21]. However, inflammatory reactions are often excessive and can damage normal tissues. Thus, the balance between neutrophil-mediated bacterial clearance and neutrophil-driven immunopathology is a critical determinant of infection outcome. Chemokine (C-X-C motif) ligand 8 (CXCL8/IL-8) can bind to G protein-coupled receptors. While CXCL8-dependent neutrophil recruitment is important for antimicrobial defense, uncontrolled CXCL8 signalling has been implicated in acute and chronic inflammatory diseases, including inflammatory lung injury [22]. CXCL8(3–72) K11R/G31P (G31P), an analogue of CXCL8 with the mutations of CXCL8 at K11 and G31 positions, is a high affinity antagonist against CXCR1 and CXCR2 [23]. Previous studies have shown that it could suppress neutrophil chemotaxis and attenuate neutrophil-dominated pulmonary inflammation in murine *Klebsiella pneumoniae* infection and a guinea pig aspiration pneumonia model, without impairing bacterial clearance [24,25]. However, G31P acts solely as an immunomodulatory agent and lacks direct antibacterial activity, leaving the pathogen burden unaddressed.

In the present study, we engineered a novel hybrid protein, G31P–BPI_233_, by fusing CXCL8(3–72) K11R/G31P to BPI_233_ through a flexible linker. We hypothesized that this fusion protein would combine two complementary activities: modulation of CXCL8-driven neutrophil recruitment and preservation of BPI-derived antibacterial and endotoxin-neutralizing effects. This study aimed to determine whether G31P–BPI_233_ could protect against CRAB infection by simultaneously enhancing antibacterial defense and limiting excessive neutrophil-associated inflammation.

## Materials and methods

### Animals and ethics statement

BALB/c mice were purchased from SPF Biotechnology Co., Ltd. (Beijing, China). BPI knockout and wild-type (WT) mice were obtained from Capital Medical University (Beijing, China) [26]. All mice were age- and sex-matched and used at 8–10 weeks of age. All animal experiments were reviewed and approved by the Institute of Biophysics, Chinese Academy of Sciences (No. ABSL-2-2023031), and were conducted in accordance with the Principles of Laboratory Animal Care. Animals were randomized to different intervention groups; however, investigators responsible for sample processing and data analysis were not blinded to group allocation. Peripheral blood from healthy volunteers was collected after written informed consent and approval by the Ethics Committee of the China–Japan Friendship Hospital (2023-KY-119).

### Bacterial strains and growth conditions

The standard bacterial strains used in this study included *A. baumannii* ATCC BAA-1605, *Escherichia coli* ATCC 35218, *E. coli* ATCC 25922, *Klebsiella pneumoniae* ATCC 700603, *Pseudomonas aeruginosa* PAO1 (ATCC BAA-47), and *P. aeruginosa* ATCC 27853, which were obtained commercially from the American Type Culture Collection (ATCC, Manassas, VA, USA). Five non-duplicate isolates of clinical CRAB (99389, 99391, 99392, 99393, and 99394) were recovered from respiratory specimens collected from patients with pneumonia at the China–Japan Friendship Hospital (Table S1). Species identification was performed using MALDI–TOF MS. Antimicrobial susceptibility testing (AST) was performed using Minimum inhibitory concentration (MIC)-based and disk-diffusion methods. MICs were determined using the VITEK 2 Compact system (bioMérieux, Marcy-l’Étoile, France) and are reported in μg/mL. Disk-diffusion testing was performed on Mueller–Hinton agar using the Kirby–Bauer method, and inhibition-zone diameters were reported in millimetres. Susceptibility categories were interpreted according to the Clinical and Laboratory Standards Institute (CLSI) M100. All strains were stored at −80°C in glycerol stocks before use. For experiments, bacteria were grown in 5 mL Luria–Bertani (LB) medium at 37°C on an orbital shaker (180 rpm). Bacterial suspensions were then adjusted to the desired concentration (e.g. ∼10⁶–10⁸ CFU/mL) using a spectrophotometer for subsequent assays. The carbapenem-resistant phenotype of *A. baumannii* ATCC BAA-1605 was confirmed in a previous study [26]. The mid-logarithmic growth phase was used for each assay.

### Protein preparation, purification, and validation

We designed and constructed the CXCL8(3–72) K11R/G31P(G31P)–BPI_233_ fusion protein, which, from the N-terminus to the C-terminus, consists of G31P, a flexible linker, and the BPI_233_ sequence elements (Figure S1A, B and Table S2). The 315-amino-acid sequence of G31P–BPI_233_ was submitted to ColabFold version 1.6.1 and modelled using the AlphaFold2-pTM monomer model. Multiple-sequence alignments were generated using MMseqs2 against UniRef and environmental sequence databases, without template information. Five models were generated with three recycles, one ensemble, and a random seed of 0. The maximum number of MSA clusters and extra sequences were set to 512 and 5,120, respectively. We turned off dropout and did not perform Amber relaxation. The resulting models were ranked according to their mean predicted local distance difference test (pLDDT) scores. The highest-ranked structure, generated using the AlphaFold2-pTM model 1, had a mean pLDDT score of 82.1 and a predicted template modelling score (pTM) of 0.610. This model was selected for visualization in PyMOL, and its per-residue pLDDT scores were used to assess local prediction confidence (Figure S1C and Figure S2). These *in silico* analyses were used to evaluate the plausibility of the intended domain organization.

The G31P–BPI_233_ sequence was cloned into the pSCm plasmid, propagated in *E. coli* JM108, and confirmed by DNA sequencing (Figure S1D). CHO-K1 cells were transfected with pSCm-G31P–BPI_233_ by electroporation (BTX ECM® 830; 360 V, 7 ms, two pulses) and selected with 30 μM methionine sulfoximine (MSX). Stable clones were screened by enzyme-linked immunosorbent assay (ELISA) and SDS–PAGE, and high-producing clones were expanded for protein production in shake flasks with in situ capture using SP Sepharose® Fast Flow resin. The resin-bound protein was purified by ion-exchange chromatography and eluted with a stepwise NaCl gradient; fractions eluted at 1.0 M NaCl were collected as the target fusion protein, buffer-exchanged, and stored at −80°C. Protein expression and preliminary identity were initially assessed by Western blot using anti-human CXCL8 antibody (Thermo Fisher Scientific, Cat# PA5-79114; 1:1000) and Alexa Fluor 680-conjugated goat anti-rabbit IgG secondary antibody (Thermo Fisher Scientific, Cat# A-21109; 1:10,000). The membrane was imaged using a gel chemiluminescence imaging analysis system (Odyssey; LI-COR Biosciences, Lincoln, NE, USA).

The recombinant BPI_233_ used in the control experiment was produced using the same expression and purification procedure as G31P–BPI_233_. G31P was chemically synthesized by QYAOBIO (ChinaPeptides, Shanghai, China) using Fmoc-based solid-phase peptide synthesis and purified by C18 reverse-phase HPLC. The expected disulfide bonds, Cys5–Cys32 and Cys7–Cys48, were introduced during peptide preparation. Peptide identity was verified by mass spectrometry, and its purity was 95% as determined by analytical HPLC. As for the control experiment, G31P, BPI_233_, and the physical G31P + BPI_233_ mixture were tested at component-equivalent molar doses relative to G31P–BPI_233_, and the mixture was freshly prepared before use.

### Serum stability immunoblotting

Purified G31P–BPI_233_ was diluted to a final concentration of 50 μg/mL in 90% (v/v) non-haemolysed, non-heat-inactivated serum obtained from naïve BALB/c mice. Samples were incubated at 37°C for 0, 2, 8, or 24 h. The 0-h sample was mixed with reducing sample buffer within 2 min of adding the fusion protein. In parallel, G31P–BPI_233_ was incubated in PBS (pH 7.4) for 0 or 24 h, and serum without added fusion protein served as a matrix blank. At the indicated endpoints, Samples were heated at 70°C for 10 min and stored at −80°C until analysis. Serum stability of G31P–BPI_233_ was assessed by Western blotting. Samples containing equivalent initial amounts of G31P–BPI_233_ were separated by SDS–PAGE alongside a Trelief Prestained Protein Ladder (Tsingke Biotechnology, Cat# TSP021) and transferred onto 0.2-μm PVDF membranes. G31P–BPI_233_ was detected using a rabbit anti-human CXCL8 polyclonal antibody (Thermo Fisher Scientific, Cat# PA5-79114; 1:1,000), followed by an HRP-conjugated mouse anti-rabbit IgG (H + L) secondary antibody (Thermo Fisher Scientific, Cat# 31464; 1:5,000). Signals were visualized using an enhanced chemiluminescence substrate under non-saturating exposure conditions.

### Endotoxin neutralization assay

Endotoxin-neutralizing activity was assessed using a chromogenic Limulus amebocyte lysate (LAL) assay, as previously described with minor revisions [27]. In brief, residual endotoxin activity was detected using the Pierce™ LAL Chromogenic Endotoxin Quantitation Kit (Thermo Scientific, Cat# 88282) according to the manufacturer's guidelines. Biotinylated LPS derived from *E. coli* O111:B4 (Invivogen, Toulouse, France) was dissolved and diluted using endotoxin-free water. The endotoxin was added to 1 µg endotoxin-free BSA to a final concentration of 0.9 EU/mL, vortexed, and then kept at room temperature for 10 min. The G31P–BPI_233_ hybrid proteins at the indicated concentrations were added to the BSA–endotoxin mixture and incubated at room temperature for 1 h. After incubation, samples were moved to endotoxin-free 96-well plates. Residual endotoxin activity was measured according to the manufacturer's instructions. The results were shown as EU/mL, and endotoxin neutralization was calculated as 100 × [1 − (EU in protein-treated sample / EU in LPS-only control)].

### Mouse pharmacokinetics study

BALB/c mice were given G31P–BPI_233_ hybrid protein by intravenous bolus injection at 10 mg/kg in a pilot pharmacokinetic study. Blood samples were collected at 0.08, 0.5, 1, 2, 4, 8, 12, and 24 h after injection (n = 3 mice at each time point). The blood collection tubes were centrifuged for 10 min at 2,000 × g at room temperature. Serum was then collected. Serum G31P–BPI233 immunoreactivity was measured using an ELISA kit (Multi Sciences, Cat# EK108) according to the manufacturer's instructions. Absorbance was measured at 450 nm. Concentrations were calculated using standard curves.

### Safety evaluation in uninfected mouse

Age- and sex-matched 8- to 10-week-old mice were administered G31P–BPI_233_ (500 μg per mouse) or saline intravenously (i.v.) (n = 5 mice per group). Clinical condition was evaluated at 0.5, 2, 6, and 24 h after administration using a predefined 0–4 scoring system: 0, normal; 1, mildly reduced activity or mild piloerection; 2, marked reduction in activity, a hunched posture, or mild respiratory abnormality; 3, severe lethargy, marked respiratory distress, or inability to move normally; and 4, moribund or dead. Blood was collected at 2 h, and serum IL-6 and TNF-α concentrations were measured using commercial ELISA kits (Mouse IL-6 ELISA Kit, Multi Sciences, Cat# EK206; Mouse TNF-α ELISA Kit, Multi Sciences, Cat# EK282). At 24 h, we collected the liver and kidney and stained them with hematoxylin and eosin (H&E) to evaluate organ injury. Blood samples were collected at 24 h, and serum was separated for biochemical analysis. Serum alanine aminotransferase (ALT), blood urea nitrogen (BUN), and creatinine (CREA) were measured on a Mindray BS-420 automated chemistry analyzer (Shenzhen Mindray Bio-Medical Electronics Co., Ltd., Shenzhen, China). BUN was quantified using a liquid dual-reagent Urea Kit (Zhongsheng Beikong Bio-Technology and Science Inc., Beijing, China). ALT and CREA were measured using an Alanine Aminotransferase Assay Kit and a Creatinine Assay Kit (Zhongsheng Beikong Bio-Technology and Science Inc., Beijing, China), respectively, according to the manufacturer's instructions.

### Dose–response experiments

Dose–response experiments were conducted as described previously [13]. In brief, bacteria were grown overnight in LB medium at 37°C and 220 rpm. Then, bacteria were diluted in saline to 1 × 10^6 CFU/mL. The bacterial suspension was mixed with recombinant G31P–BPI_233_ hybrid protein at 1.95, 3.9, 7.8, 15.625, 31.25, 62.5, 125, and 250 μg/mL. The mixture was incubated for 1 h at 37°C without shaking. After that, bacteria were serially diluted and plated on LB agar plates. The plates were incubated overnight at 37°C. Colony-forming units were counted the next day.

### Neutrophil isolation and purification

For human neutrophils, fresh blood samples (12 mL) were collected from healthy volunteers. The blood was carefully layered onto Histopaque-1077 (Sigma-Aldrich, Cat# 10771) over Histopaque-1119 (Sigma-Aldrich, Cat# 11191). Samples were centrifuged for 30 min at 340 × g at room temperature with no brake or acceleration. Neutrophils at the layer between Histopaque-1077 and Histopaque-1119 were carefully collected. The isolated neutrophils were washed twice with DPBS. Then, the cells were resuspended in complete RPMI 1640 medium.

Mouse bone marrow (BM) neutrophils were isolated by discontinuous Percoll density-gradient centrifugation. Briefly, femurs and tibias were harvested, and BM cells were flushed with cold PBS. Single-cell suspensions were prepared by gentle pipetting and filtration through a 70-μm cell strainer. Red blood cells were lysed using a Red Blood Cell Lysis Buffer (Invitrogen, Cat# 00-4333-57). Discontinuous Percoll gradients were prepared by diluting Percoll (Yeasen, Cat# 40501ES60) in PBS to 55%, 65%, and 75%. Cell suspensions were layered onto the gradients and centrifuged at 1,500 × g for 30 min at room temperature without a break. Neutrophils were collected from the interface between the 65% and 75% layers, washed, and resuspended in complete RPMI 1640 medium for subsequent experiments.

Neutrophil purity was assessed by flow cytometry (Figure S3). Human neutrophils were identified as CD45⁺CD16⁺CD66b⁺ cells using the following antibodies: anti-human CD45 (BioLegend, Cat# 368526), anti-human CD16 (BioLegend, Cat# 980102), and anti-human CD66b (BioLegend, Cat# 305108) [28], and mouse BM neutrophils as CD45⁺Ly6G⁺CD11b⁺ cells using the following antibodies: anti-CD11b (Miltenyi Biotec, Cat# 130-113-805), anti-Ly6G (Miltenyi Biotec, Cat# 130-119-126), and anti-CD45 (Miltenyi Biotec, Cat# 130-110-803) [29]. The proportion of isolated neutrophils exceeded 80%, indicating that cells of this purity and viability were suitable for subsequent functional validation assays.

### Neutrophil chemotaxis assay

Neutrophil chemotaxis was assessed using a Transwell migration assay (LABSELECT, Cat# 14322), based on a modified Boyden chamber method. Neutrophils isolated from human peripheral blood were suspended at 1 × 10^6 cells/mL in 100 μL PBS and added to the upper chamber. The lower chamber contained PBS alone, CXCL8 (20 ng/mL), or CXCL8 in combination with the indicated protein treatment. The chambers were incubated for 40 min at 37°C in a humidified atmosphere containing 5% CO₂. For the dose-comparison experiment, the lower chambers contained PBS, CXCL8 (20 ng/mL) alone, or CXCL8 combined with G31P–BPI_233_ at 20 or 200 ng/mL. Following incubation, neutrophils that had migrated to the lower surface of the membrane were fixed and stained with Wright–Giemsa stain. At least five randomly selected, non-overlapping fields per insert were imaged under a 100× oil-immersion objective. Migrated neutrophils were counted, and the mean number of migrated cells per field was calculated for each insert. For the head-to-head comparator experiment, CXCL8-induced neutrophil migration was examined in the presence of PBS, CXCL8 (20 ng/mL) alone, or CXCL8 (20 ng/mL) supplemented with G31P–BPI_233_ fusion protein (20 ng/mL) or equimolar noncovalent G31P (4.78 ng/mL), BPI_233_ (14.79 ng/mL), or a mixture of G31P and BPI_233_. Following incubation, the cell suspension in each lower chamber was collected. Cells that had settled or adhered to the bottom of the lower well were recovered by gentle resuspension and rinsing with PBS. The collected fractions were pooled and adjusted to the same final volume. The number of neutrophils that migrated to the bottom chamber was counted using a Scepter™ 2.0 Handheld Automated Cell Counter (Millipore Ltd, ON, Canada) following trypan blue exclusion [30]. Each recovered sample was measured in triplicate. The total number of migrated neutrophils per insert was calculated by multiplying the measured cell concentration by the final recovery volume. For both experiments, the chemotactic index (CI) was calculated relative to spontaneous migration in the PBS control as follows: CI = *N*_condition_/*N*_PBS_.

### Stimulation of peripheral blood mononuclear cells (PBMCs)

Human PBMCs were isolated as described previously [13]. In detail, freshly obtained blood (12 mL) from healthy volunteers was collected in heparinized tubes (BD Vacutainer, Cat# 367886). The blood was centrifuged in Leucosep tubes containing Ficoll-Paque PLUS (Cytiva, Cat# 17-1440-02) at 1000 × g for 10 min. Leukocytes were collected from the interphase and subsequently washed twice. The PBMC pellet was resuspended in complete RPMI 1640 medium, and 1 × 10^5 PBMCs were seeded into a 96-well plate and incubated for 3 h before stimulation. *E. coli* O111:B4-derived LPS was diluted in complete RPMI 1640 medium to 10 ng/mL. For each donor, the following conditions were tested in parallel: unstimulated PBMCs receiving medium alone, PBMCs stimulated with LPS plus vehicle, and PBMCs stimulated with LPS in combination with G31P, BPI_233_, an equimolar noncovalent mixture of G31P and BPI_233_, or the G31P–BPI_233_ fusion protein. The protein comparison was performed at a fusion-equivalent concentration of 50 μg/mL. The mass concentrations of G31P and BPI_233_ were adjusted according to their respective molecular masses to provide the same molar exposure to each active domain as that provided by the fusion protein. After 24 h of stimulation, cell-culture supernatants were collected and stored at −20°C until cytokine analysis.

### Mouse septicemia infection model and treatment

Septicemia was induced in age- and sex-matched 8- to 10-week-old mice by an intraperitoneal inoculation of a bacterial suspension of CRAB ATCC BAA-1605 at a challenge dose of 5 × 10^6 CFU per mouse. Mice were assigned to receive either sterile saline (n = 15) or G31P–BPI_233_ (500 μg per mouse; n = 10) by intraperitoneal administration 1 h after infection. Mouse survival was followed over 3 days.

### Mouse lung infection model and treatment

Age- and sex-matched 8- to 10-week-old mice were challenged intranasally (i.n.) with the indicated dose of CRAB (5 × 10^7 CFU) after anesthesia with Avertin. For treatment experiments, mice were randomly assigned to six groups (n = 5 per group): saline, G31P alone, BPI_233_ alone, an equimolar noncovalent G31P + BPI_233_ mixture, G31P–BPI_233_, or colistin. At 1 h post-infection, mice received a single treatment of saline (200 μL, i.v.), G31P (119.4 μg per mouse, i.v.), BPI_233_ (369.7 μg per mouse, i.v.), the G31P + BPI_233_ mixture (119.4 μg G31P plus 369.7 μg BPI_233_ per mouse, i.v.), or G31P–BPI_233_ (500 μg per mouse, i.v.). All protein preparations were adjusted to a final injection volume of 200 μL. Colistin sulfate was administered intraperitoneally (i.p.) at 8 mg/kg, with the injection volume adjusted according to body weight. No additional doses were given. Mice were euthanized at 24 h post-infection for sample collection. For BPI^−/−^ and WT time-course experiments, animals were infected as described above and euthanized at 1, 2, or 3 days post-infection (dpi), with five mice per genotype analysed at each time point (Bacterial burden and serum endotoxin measurements included four mice per genotype per time point, whereas flow-cytometric analyses included five). Uninfected control mice received intranasal saline instead of bacterial challenge and were treated with saline (n = 3).

### Quantification of cytokines

Levels of human IL-6 and TNF-α in human cell culture supernatants or murine BALF were quantified by ELISA using commercial kits according to the manufacturer's instructions (Human IL-6 ELISA Kit, Multi Sciences, Cat# EK106; Human TNF-α ELISA Kit, Multi Sciences, Cat# EK182; Mouse IL-6 ELISA Kit, Multi Sciences, Cat# EK206; Mouse TNF-α ELISA Kit, Multi Sciences, Cat# EK282). BALF was collected by cannulating the trachea and gently lavaging the lungs with 1 mL sterile PBS three times. The recovered lavage fluid was pooled for each mouse (typically 2–3 mL), kept on ice, and centrifuged at 400 × g for 7 min at 4°C. Standards and samples were added to pre-coated 96-well plates, followed by incubation with detection antibodies and streptavidin–HRP. The reaction was developed using tetramethylbenzidine (TMB) substrate and stopped with the stop solution. Absorbance was measured at 450 nm using a microplate reader. Cytokine concentrations were calculated from standard curves.

### Hematoxylin and eosin staining

The right lung tissues were fixed with 4% paraformaldehyde, embedded, sectioned, and stained with hematoxylin and eosin (H&E) to evaluate inflammatory injury. The sections were observed using a Panoramic SCAN 150 (3DHISTECH, Budapest, Hungary). Five fields of view were randomly selected from whole-slide images for each mouse and then analysed using CaseViewer software (3DHISTECH). A lung histopathology score was determined using a semiquantitative scoring system [31].

### Bacterial colonization and endotoxin measurement

The left lung and spleen tissues were collected aseptically at 24 h post-infection, weighed, and homogenized in 1 mL of PBS. Tissue homogenates were serially diluted and plated on LB agar for CFU enumeration, and results were expressed as log10 (CFU/g tissue). A time-course experiment was conducted with groups of BPI^-/-^ and WT mice. At 1, 2, and 3 dpi, the left lung and spleen were collected. Tissue homogenates were serially diluted and plated on LB for CFU enumeration. At the indicated time points, blood samples were also collected, and serum endotoxin activity was measured in EU/mL using a chromogenic LAL assay (Pyrochrome, Cat# C1500) according to the manufacturer's instructions.

### Flow cytometric analysis of neutrophil phenotype and maturation

The whole lung and blood were collected aseptically at 24 h post-infection for treatment experiments, and samples from BPI^-/-^ and WT mice were collected at 1, 2, and 3 dpi to assess neutrophil changes. BM was collected from BPI^-/-^ and WT mice at 1, 2, and 3 dpi to explore neutrophil differentiation and maturation.

For phenotypic analysis, neutrophils in blood and lung were identified as singlet live CD45⁺CD11b⁺Ly6G⁺ cells using the antibodies described above. For neutrophil maturation analysis, BM single-cell suspensions were prepared and stained with the following antibodies: anti-CD115 (Miltenyi Biotec, Cat# 130-112-828), anti-Gr-1 (Miltenyi Biotec, Cat# 130-119-794), anti-CXCR4 (Miltenyi Biotec, Cat# 130-127-483), anti-CXCR2 (Miltenyi Biotec, Cat# 130-115-639), anti-Siglec-F (Miltenyi Biotec, Cat# 130-112-333), and anti-cKit (BioLegend, Cat# 135124). Neutrophil subsets were defined as follows: preneutrophils (CD115^−^ Siglec-F^−^ Gr-1⁺ CD11b⁺ cKit^int CXCR4⁺), immature neutrophils (CD115^−^ Siglec-F^−^ Gr-1⁺ CD11b⁺ cKit^low/neg CXCR4^−/low Ly6G^low/+ CXCR2^−^), and mature neutrophils (CD115^−^ Siglec-F^−^ Gr-1⁺ CD11b⁺ cKit^low/neg CXCR4^−/low Ly6G⁺ CXCR2⁺) [32].

Data were acquired using a BD FACSymphony with Diva Version 8.0 software. Data analysis was conducted using FlowJo Version 10.4.0 (TreeStar Inc., Ashland, OR, USA).

### Neutrophil-mediated bactericidal assay

For neutrophil killing assays, neutrophils isolated from human blood were adjusted to 1 × 10^7 cells/mL in complete RPMI 1640 medium. Neutrophils (1 × 10^6 cells) were seeded into 96-well plates and preincubated for 30 min. For the corresponding neutrophil-free conditions, we added an equal volume of complete RPMI 1640 medium instead of the cell suspension. Opsonized CRAB (4 × 10^7 CFU; MOI = 40) was then added. For the concentration–response experiment, CRAB was incubated with the indicated concentrations of the recombinant G31P–BPI_233_ fusion protein in the presence or absence of neutrophils. For the head-to-head protein comparison, the following treatments were tested in parallel: G31P, BPI_233_, an equimolar noncovalent mixture of G31P and BPI_233_, and the G31P–BPI_233_ fusion protein. Each treatment was evaluated both in the absence and presence of neutrophils. The comparison was performed at a fusion-equivalent concentration of 3.9 μg/mL, with the comparator doses adjusted to provide equimolar exposure to the corresponding active domains. After incubation for 2 h, neutrophils in the corresponding wells were lysed with 0.1% Triton X-100 on ice for 15 min. Viable bacteria were quantified by plating serial dilutions onto LB agar plates. Wells containing CRAB and vehicle without neutrophils were used as the reference control. The bactericidal rate was calculated as: $K_{p,\pm N}(\%)=\frac{\mathrm{CF}U_{\mathrm{vehicle},-N}-\mathrm{CF}U_{p,\pm N}}{\mathrm{CF}U_{\mathrm{vehicle},-N}}\times \;100$, where *CFU*_vehicle,-N_ represents the viable bacterial count in the vehicle-treated, neutrophil-free control, and *CFU*_p,±N_ represents the viable bacterial count following treatment p, either with or without neutrophils.

### Serum bactericidal assay

For the serum-mediated bactericidal assay, 50 µL of a CRAB suspension was combined with 100 µL of pre-warmed human serum. For the corresponding serum-free conditions, an equal volume of serum-free assay medium was added instead of human serum. For the concentration–response experiment, CRAB was incubated with the indicated concentrations of recombinant G31P–BPI_233_ fusion protein in the presence or absence of human serum. For the head-to-head protein comparison, vehicle, G31P, BPI_233_, an equimolar noncovalent mixture of G31P and BPI_233_, and the G31P–BPI_233_ fusion protein were tested in parallel. Each treatment was evaluated both in the absence and presence of human serum. The comparison was performed at a final fusion-equivalent concentration of 3.9 μg/mL, with the comparator doses adjusted to provide equimolar exposure to the corresponding active domains. After incubation for 2 h at 37°C, samples were serially diluted in sterile PBS and plated on LB agar plates. Viable bacterial counts were determined by colony enumeration and expressed as CFU per well. Wells containing CRAB and vehicle without human serum were used as the reference control. The bactericidal rate was then calculated as mentioned above.

### Bead uptake assay with fluorescent microspheres

Neutrophils purified from the BM of WT/BPI^−/−^ mice were resuspended in complete RPMI 1640 medium and preincubated for 30 min. Human peripheral blood neutrophils were preincubated with medium or recombinant G31P–BPI_233_ hybrid protein for 30 min at 37°C. The cells were then treated with 100 nM PMA (Sigma-Aldrich) or CRAB (MOI = 20), or left untreated, for another 30 min at 37°C. Then, fluorescent microspheres (Sigma, Cat# L303) were added at a final concentration of 0.015% (v/v). The co-culture was incubated for another 30 min. Uptake was stopped by placing the cells on ice. The cells were then washed to remove beads that were not taken up. Bead uptake was measured by flow cytometry and shown as the percentage of bead-positive neutrophils.

### Quantification of NET-associated myeloperoxidase (MPO) and extracellular double-stranded DNA (dsDNA) markers

For *in vivo* analysis, lung tissues were collected from infected mice at the indicated time points. The tissues were homogenized in cold PBS. Then, the samples were centrifuged to remove cell debris. The lung homogenate supernatants were collected for MPO and dsDNA measurement. MPO levels were measured using a mouse MPO ELISA kit (Multi Sciences, Cat# EK2133). dsDNA levels were measured using the Quant-iT PicoGreen dsDNA assay kit (Invitrogen, Cat# P7589) [33,34]. For *in vitro* analysis, human neutrophils were prepared and treated as described above. Cell-free supernatants were collected. MPO release was measured using a human MPO ELISA kit (Multi Sciences, Cat# EK1133). dsDNA was measured using the Quant-iT PicoGreen dsDNA assay kit (Invitrogen, Cat# P7589) according to the manufacturer's instructions.

### Assessment of neutrophils apoptosis

Neutrophil apoptosis was measured using an FITC Annexin V Apoptosis Detection Kit (BD Pharmingen, Cat# 556547) according to the manufacturer's instructions. The samples were then analysed by flow cytometry. For *in vivo* experiments, lung single-cell suspensions were prepared from WT and BPI^−/−^ mice before infection or at 2 days post-infection (dpi). Neutrophils were defined as CD45⁺Ly6G⁺CD11b⁺ cells. Apoptosis was analysed using Annexin V and PI staining. For *in vitro* mouse experiments, neutrophils were isolated from the bone marrow of WT or BPI^−/−^ mice. The cells were treated with LPS or CRAB for 8 h at 37°C. Then, Annexin V/PI staining and flow cytometry were performed. For human neutrophils, peripheral blood neutrophils were preincubated with medium or recombinant G31P–BPI_233_ for 30 min at 37°C, followed by stimulation with LPS or CRAB for 8 h at 37°C. Human neutrophils were identified as CD45⁺CD16⁺CD66b⁺ cells as described above, except that the anti-human CD16 antibody (BioLegend, Cat# 302032) was replaced to avoid spectral overlap, and then stained with Annexin V and PI and analysed by flow cytometry. Early apoptotic cells were defined as Annexin V⁺PI^−^, and late apoptotic or necrotic cells were defined as Annexin V⁺PI⁺.

### Statistical analyses

Statistical analyses were performed using GraphPad Prism version 10.2.2. Data were compared between two groups using Student's t-test, and between more than two groups using one-way analysis of variance. Mouse survival curves were analysed using log-rank analysis for the Kaplan–Meier test. All data are presented as mean ± SEM unless otherwise indicated, as appropriate. *P* < 0.05 was considered statistically significant, with multiple comparisons adjusted where applicable.

## Results

### Generation and characterization of the G31P–BPI_233_ fusion protein

G31P–BPI_233_ was made by joining CXCL8(3–72)K11R/G31P to the BPI_233_ domain with a flexible linker. Sequence analysis confirmed the designed protein structure and the introduced mutations (Figure S1A–C). AlphaFold2/ColabFold modelling showed a likely two-domain structure linked by a flexible linker. The confidence was lower in the linker and terminal regions (Figures S1C and S2). G31P–BPI_233_ was expressed in CHO-K1 cells and purified by cation-exchange chromatography. A main elution peak appeared at the expected position (Figure 1A). Western blotting with an anti-CXCL8 antibody showed a major band at the expected molecular weight (Figure 1B). We further examined the stability of G31P–BPI_233_ under physiologically relevant serum conditions. Following incubation in 90% non-heat-inactivated mouse serum at 37 °C, a reactive band co-migrating with the 0-h fusion-protein species remained detectable at 2, 8, and 24 h. A corresponding fusion-protein-sized band was also detected after incubation in PBS for 24 h (Figure S4). These findings indicate that a full-length-sized species persisted for at least 24 h under the tested serum conditions.

**Figure 1.**
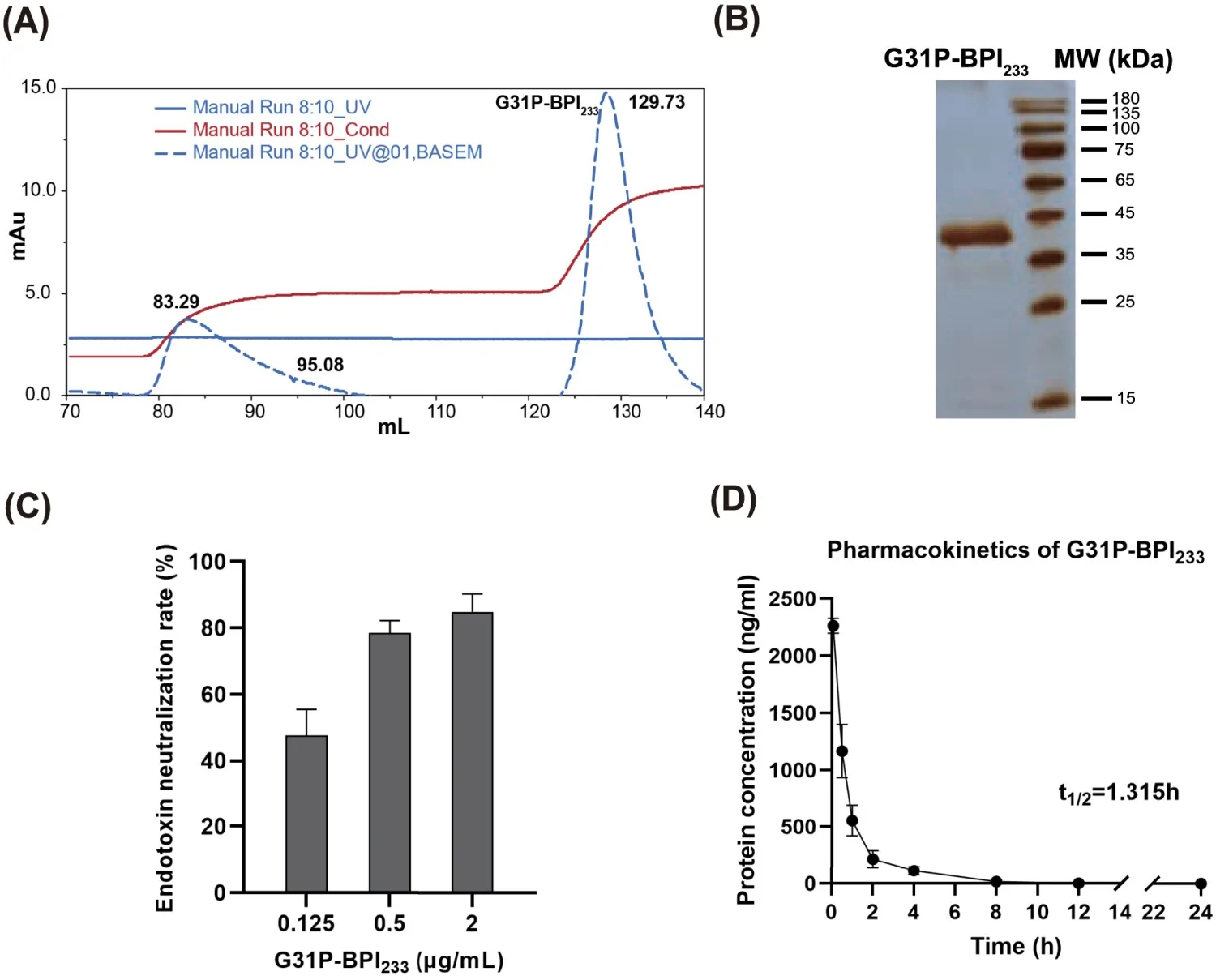
Expression, purification, endotoxin-neutralizing activity, and pharmacokinetic profile of G31P–BPI_233._ (A) Representative cation-exchange chromatography profile showing purification of recombinant G31P–BPI_233_. (B) Western blot analysis of purified G31P–BPI_233_. (C) Endotoxin-neutralizing activity of G31P–BPI_233_ was evaluated using the LAL assay. (D) Pharmacokinetic profile of G31P–BPI_233_ after intravenous administration in mice (n = 3 mice at each time point). Serum protein concentrations were measured at the indicated time points. Data are presented as mean ± SEM.

In the chromogenic LAL assay, G31P–BPI233 reduced residual endotoxin activity in a dose-dependent way (Figure 1C). In the pilot pharmacokinetic study, circulating immunoreactivity decreased quickly after intravenous injection. The apparent half-life was about 1.3 h (Figure 1D). To evaluate the basic safety of G31P–BPI_233_, uninfected BALB/c mice received a therapeutic intravenous dose of G31P–BPI_233_ or saline and were observed for 24 h. One of five G31P–BPI233-treated mice showed a transient clinical score of 1 at 0.5 h, which returned to 0 by 2 h, whereas all other mice maintained a score of 0 throughout the observation period (Figure S5A). Serum IL-6 and TNF-α concentrations measured at 2 h were comparable between groups (Figure S5B, C). At 24 h, serum ALT, BUN, and CREA also did not differ detectably between the saline and G31P–BPI_233_ groups (Figure S5D-F). In addition, histological analysis showed that G31P–BPI_233_ did not cause drug-induced liver or kidney damage (Figure S5G, H). Thus, these findings support preliminary tolerability over 24 h, with a transient mild clinical abnormality in one mouse.

### G31p–BPI_233_ exhibits dose-dependent bactericidal activity against reference strains and clinical CRAB isolates *in vitro*.

To test the direct antibacterial activity of the fusion protein, dose–response assays were performed against representative Gram-negative bacteria, including *E. coli, P. aeruginosa, K. pneumoniae,* and *A. baumannii*, together with five clinical CRAB isolates. The heatmap in Figure 2(A) summarizes the bactericidal profiles across the complete strain panel. G31P–BPI_233_ produced concentration-dependent killing of all tested strains. At 125–250 μg/mL, it achieved near-complete killing of most reference strains, whereas *K. pneumoniae* ATCC 700603 was less susceptible across the tested concentration range. The five clinical isolates were resistant to imipenem and meropenem and exhibited distinct phenotypic antimicrobial susceptibility profiles (Table S1). All five isolates showed concentration-dependent susceptibility to G31P–BPI_233_, with near-complete killing observed at 31.25-62.5 μg/mL (Figure 2A and Figure S6G–K). Interestingly, at matched concentrations, these isolates were generally more susceptible than the CRAB reference strain *A. baumannii* ATCC BAA-1605. Complete strain-specific dose–response plots, including data from individual biological replicates, are provided in Figure S6.

**Figure 2.**
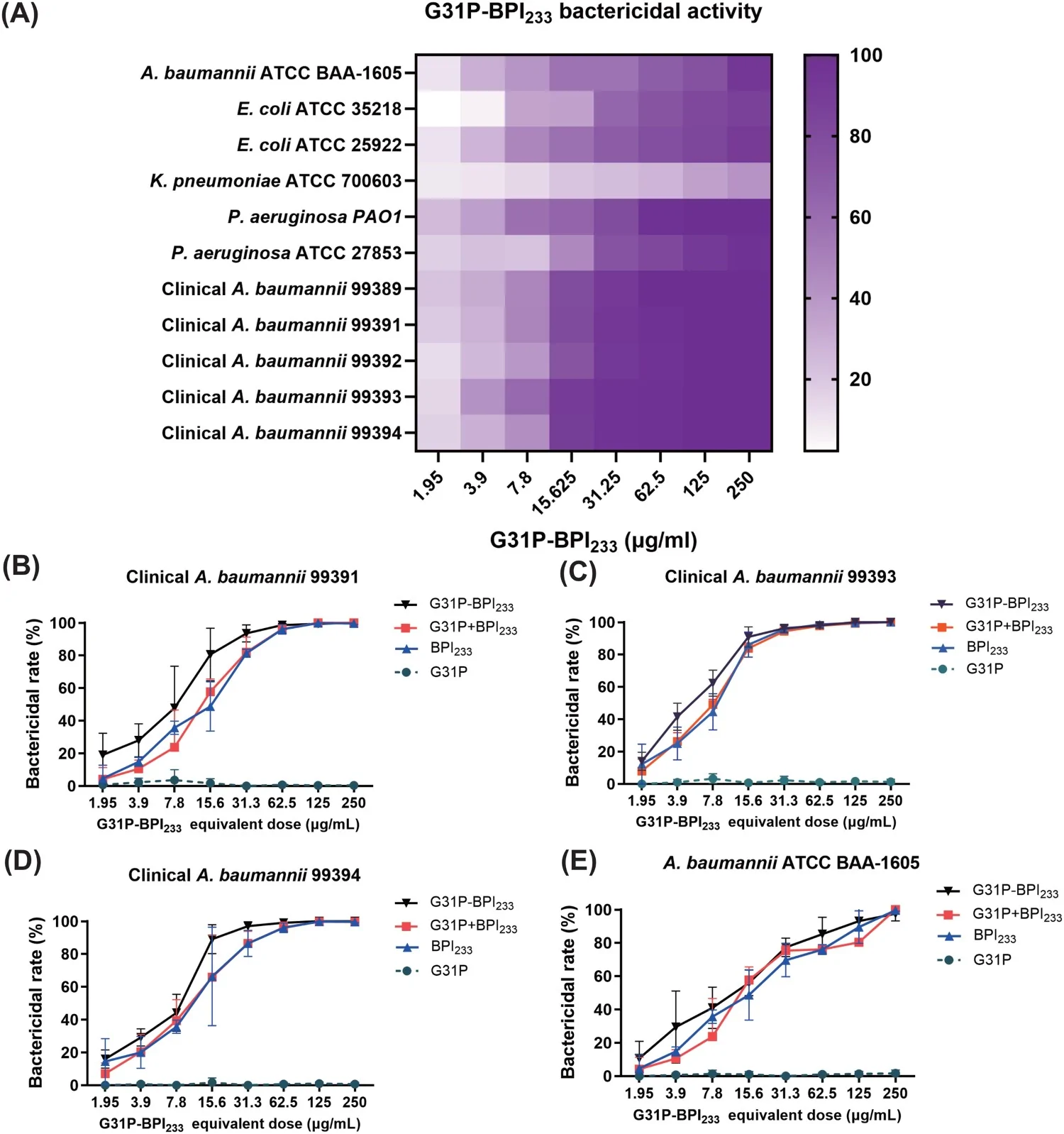
Dose-dependent bactericidal activity of G31P–BPI_233_. (A) Heatmap summarizing the mean bactericidal rates against six reference strains and five clinical CRAB isolates. (B–E) Dose–response comparison of G31P–BPI_233_, a noncovalent G31P + BPI_233_ mixture, BPI_233_ alone, and G31P alone against clinical *A. baumannii* isolates 99391 (B), 99393 (C), and 99394 (D), and *A. baumannii* ATCC BAA-1605 (E). Component doses were molar-matched to the corresponding fusion-protein doses. Data are presented as mean ± SD.

To determine whether covalent fusion affected direct bactericidal activity, we compared G31P–BPI_233_ with G31P alone, BPI_233_ alone, and a noncovalent G31P + BPI_233_ mixture at component-equivalent molar doses. These comparisons were performed against *A. baumannii* ATCC BAA-1605 and three representative clinical isolates, 99391, 99393, and 99394. G31P alone showed little or no direct bactericidal activity, whereas BPI_233_ alone and the noncovalent mixture retained measurable activity. Across the four tested *A. baumannii* strains, no significant differences in bactericidal activity were detected among G31P–BPI_233_, BPI_233_ alone, and the noncovalent G31P + BPI_233_ mixture (Figure 2B–E). In contrast, G31P alone showed little or no direct bactericidal activity. These findings indicate that G31P–BPI_233_ retained the BPI_233_-associated bactericidal function after fusion *in vitro.*

### G31p–BPI_233_ inhibited CXCL8-induced neutrophil chemotaxis

We next assessed the effects of G31P–BPI_233_ and its individual components on CXCL8-induced neutrophil migration using a Transwell assay. G31P–BPI_233_ at 20 and 200 ng/mL significantly reduced the chemotactic index compared with CXCL8 alone (Figure 3A; representative images in Figure S7). At a molar-matched dose, G31P alone and the G31P + BPI_233_ mixture also inhibited neutrophil migration, whereas BPI_233_ alone showed little inhibitory activity (Figure 3B). These findings indicate that G31P–BPI_233_ retains the CXCL8-antagonizing activity of its G31P component following fusion.

**Figure 3.**
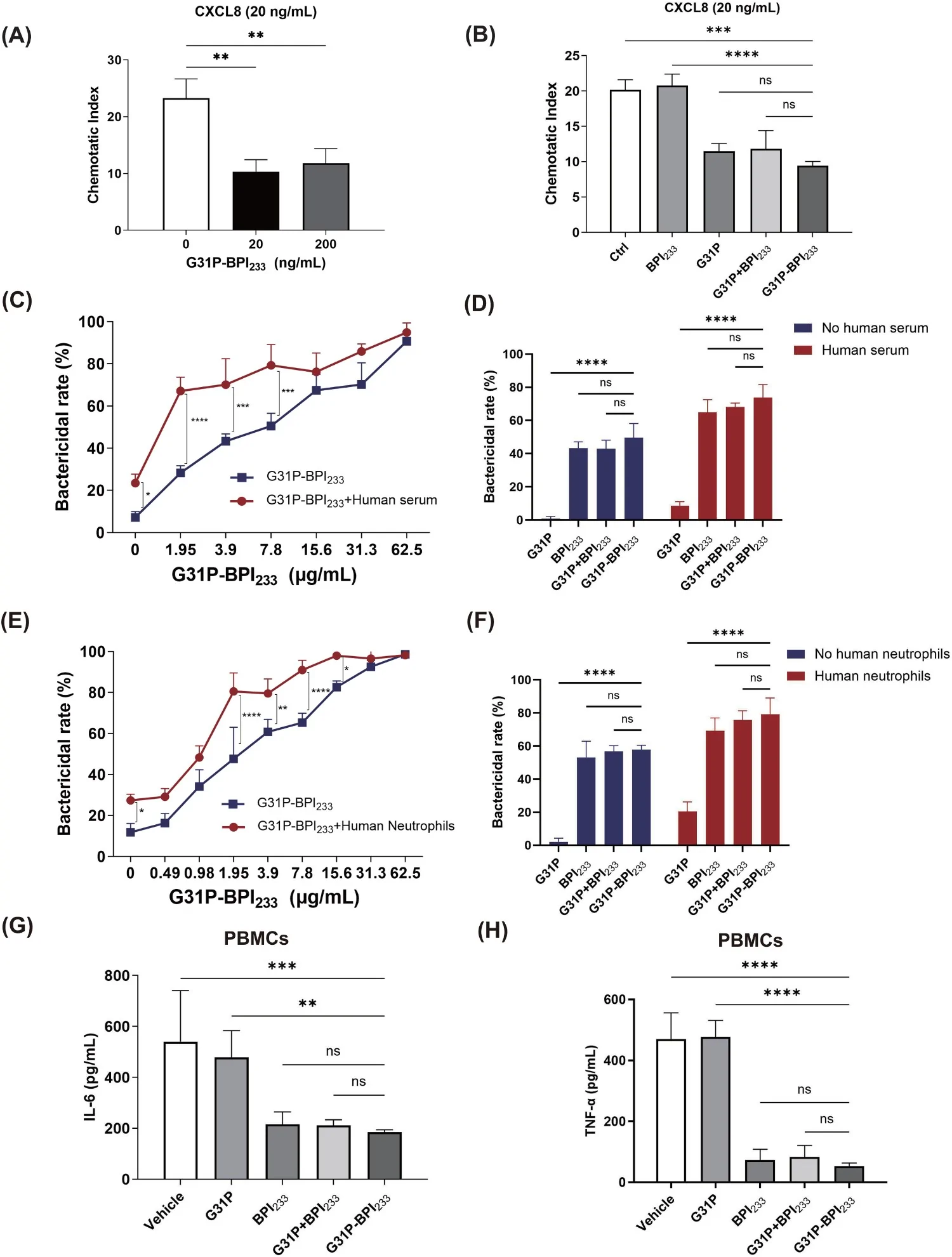
G31P–BPI_233_ retains G31P-mediated inhibition of neutrophil chemotaxis and BPI_233_-associated antibacterial and anti-inflammatory activities. (A) CXCL8-induced human neutrophil chemotaxis in the presence of 0, 20, or 200 ng/mL G31P–BPI_233_. Representative images are shown in Figure S7. (B) Comparison of G31P–BPI_233_ with G31P, BPI_233_, and the noncovalent G31P + BPI_233_ mixture at a fusion-equivalent dose of 20 ng/mL. (C, D) Concentration–response and protein-comparison assays of bactericidal activity against CRAB in the absence or presence of human serum. (E, F) Corresponding assays in the absence or presence of human neutrophils. Protein comparisons in (D) and (F) were performed at a fusion-equivalent dose of 3.9 μg/mL. (G, H) IL-6 and TNF-α concentrations in supernatants from LPS-stimulated human peripheral blood mononuclear cells treated with the indicated preparations at a fusion-equivalent dose of 50 μg/mL. Component doses were molar-matched to the corresponding fusion-protein doses. Data are presented as mean ± SEM. ns, not significant; *P < 0.05; **P < 0.01; ***P < 0.001; ****P < 0.0001.

### G31p–BPI_233_ cooperated with serum- and neutrophil-mediated bactericidal activity *in vitro*

We further tested whether G31P–BPI_233_ could increase serum- and neutrophil-mediated bacterial killing *in vitro*. Human serum alone and human neutrophils alone showed mild bacterial killing, with killing rates of about 23% and 27%, respectively. In the serum bactericidal assay, G31P–BPI_233_ alone killed CRAB in a dose-dependent way. The addition of human serum further increased the killing rate at the tested concentrations. Significant increases were observed at 1.95, 3.9, and 7.8 μg/mL G31P–BPI_233_ (Figure 3C). In the neutrophil killing assay, G31P–BPI_233_ combined with human neutrophils showed stronger bacterial killing than G31P–BPI_233_ alone. Significant differences were observed at 1.95, 3.9, 7.8, and 15.6 μg/mL (Figure 3E). At higher concentrations, mainly 31.3–62.5 μg/mL, bacterial killing reached near-complete levels in the combination group.

At a molar-matched dose equivalent to 3.9 μg/mL G31P–BPI_233_, G31P alone showed little direct bactericidal activity. In contrast, BPI_233_, the G31P + BPI_233_ mixture, and G31P–BPI_233_ produced comparable killing, both with and without human serum or neutrophils (Figure 3D, F). No significant differences were detected among these three BPI_233_-containing treatments. These results indicate that the fusion protein retains the bactericidal activity associated with its BPI_233_ component and remains active in the presence of human innate immune effectors.

### Anti-inflammatory activity of G31P–BPI_233_ in human PBMCs

To evaluate its anti-inflammatory activity, LPS-stimulated human PBMCs were treated with G31P, BPI_233_, an equimolar G31P + BPI_233_ mixture, or G31P–BPI_233_. G31P alone had little effect on LPS-induced cytokine production. In contrast, BPI_233_, the G31P + BPI_233_ mixture, and G31P–BPI_233_ reduced the secretion of IL-6 and TNF-α (Figure 3G, H). The effects of G31P–BPI_233_ did not differ significantly from those of BPI_233_ alone or the mixture. These findings indicate that the fusion protein retains the BPI_233_-associated ability to suppress LPS-induced inflammatory responses.

### G31p–BPI_233_ protected mice against CRAB septicemia and pulmonary infection *in vivo*

To test the therapeutic effect of G31P–BPI_233_ *in vivo*, we first built a septicemia model by intraperitoneal infection with CRAB. Saline-treated mice died quickly after 1 dpi. However, mice treated with G31P–BPI_233_ were fully protected, with 100% survival maintained at 3 dpi (Figure 4A). We next compared G31P–BPI_233_ with G31P, BPI_233_, an equimolar G31P + BPI_233_ mixture, and the standard antibiotic colistin in the CRAB pneumonia model. At 24 h post-infection, saline-treated mice exhibited extensive inflammatory infiltration, alveolar disruption, and severe lung injury. G31P–BPI233 significantly reduced histological lung injury scores compared with saline treatment (Figure 4B, C). Lung injury scores did not differ significantly between the G31P–BPI233 and colistin groups (Figure 4C). G31P–BPI_233_ also significantly reduced pulmonary IL-6 and TNF-α levels compared with saline treatment (Figure 4D, E). Notably, the fusion protein produced greater reductions in these inflammatory mediators than BPI_233_ alone or the G31P + BPI_233_ mixture. BALF IL-6 levels were also significantly lower in the G31P–BPI_233_ group than in the colistin group (Figure 4D). Consistent with its bactericidal activity, G31P–BPI_233_ significantly reduced lung bacterial burden and pulmonary endotoxin levels compared with saline treatment (Figure 4F, G). Both endpoints were numerically lower in the G31P–BPI_233_ group than in the equimolar G31P + BPI_233_ mixture group, although the between-group differences did not reach statistical significance. No significant differences in lung bacterial burden or pulmonary endotoxin activity were detected between the G31P–BPI_233_ and colistin groups (Figure 4F, G). Nevertheless, among the tested treatments, the fusion protein showed the most consistent numerical improvement across bacterial, inflammatory, and histopathological outcomes.

**Figure 4.**
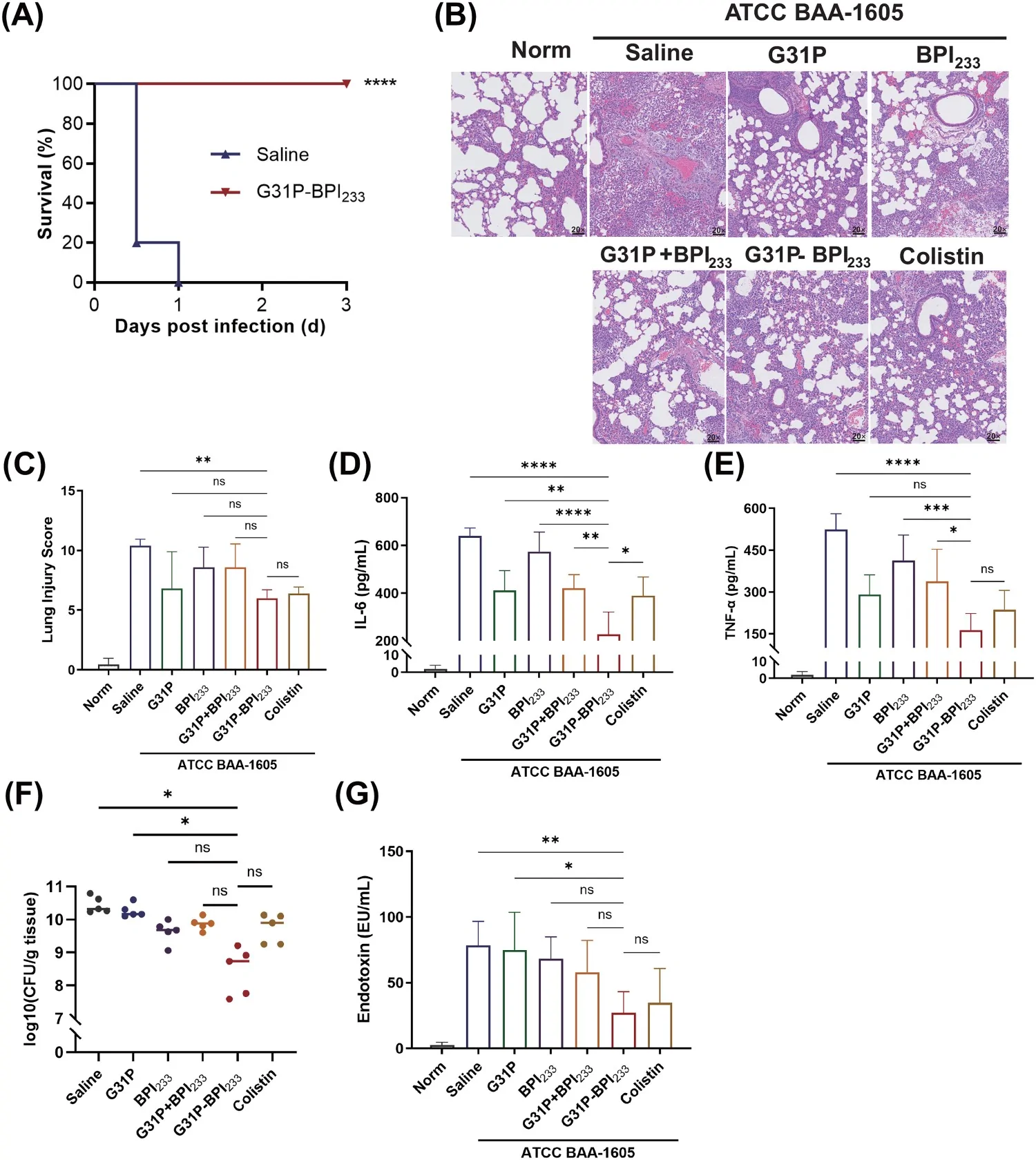
G31P–BPI_233_ protected mice against carbapenem-resistant *Acinetobacter baumannii* infection. (A) Survival of mice with lethal CRAB septicemia treated with saline (n = 15) or G31P–BPI_233_ (n = 10). (B–G) Mice were intranasally challenged with *A. baumannii* ATCC BAA-1605 and treated with saline, G31P, BPI_233_, an equimolar G31P + BPI_233_ mixture, G31P–BPI_233_, or colistin (n = 5 per group). Uninfected mice served as normal controls (n = 3). Samples were collected 24 h after infection. (B) Representative lung H&E staining; original magnification, 20×. (C) Lung injury scores. (D, E) IL-6 and TNF-α levels in bronchoalveolar lavage fluid. (F) Lung bacterial burden expressed as log10 CFU/g tissue. (G) Endotoxin activity in lung homogenates. Component doses were molar-matched to the corresponding fusion-protein dose. Data are presented as mean ± SEM. ns, not significant; *P < 0.05; **P < 0.01; ***P < 0.001; ****P < 0.0001.

Together, these findings demonstrate robust *in vivo* activity of G31P–BPI_233_ against CRAB infection. The fusion protein improved short-term survival in septicemic mice and concurrently reduced bacterial burden, endotoxin levels, pulmonary inflammation, and lung injury in the pneumonia model.

### G31p–BPI_233_ reduced neutrophil recruitment and NET-associated markers during CRAB pulmonary infection

To determine whether G31P–BPI_233_ altered neutrophil-associated inflammatory responses during CRAB pneumonia, we quantified pulmonary and circulating neutrophils by flow cytometry. Compared with saline treatment, G31P–BPI_233_ significantly reduced the percentages of pulmonary and circulating neutrophils (Figure 5A–D). The pulmonary neutrophil percentage was also lower following G31P–BPI_233_ treatment than following BPI_233_ treatment. However, it did not differ significantly from the percentages observed with G31P, the equimolar mixture, or colistin. Pulmonary MPO and extracellular dsDNA levels were also elevated after CRAB infection (Figure 5E, F). G31P–BPI_233_ significantly reduced both markers compared with saline treatment. MPO levels were also lower than those following BPI_233_ treatment, whereas dsDNA levels were lower than those following the equimolar mixture. Neither marker differed significantly between G31P–BPI_233_ and colistin. Collectively, these findings indicate that G31P–BPI_233_ modestly limited neutrophil accumulation and neutrophil-associated inflammation during CRAB pneumonia.

**Figure 5.**
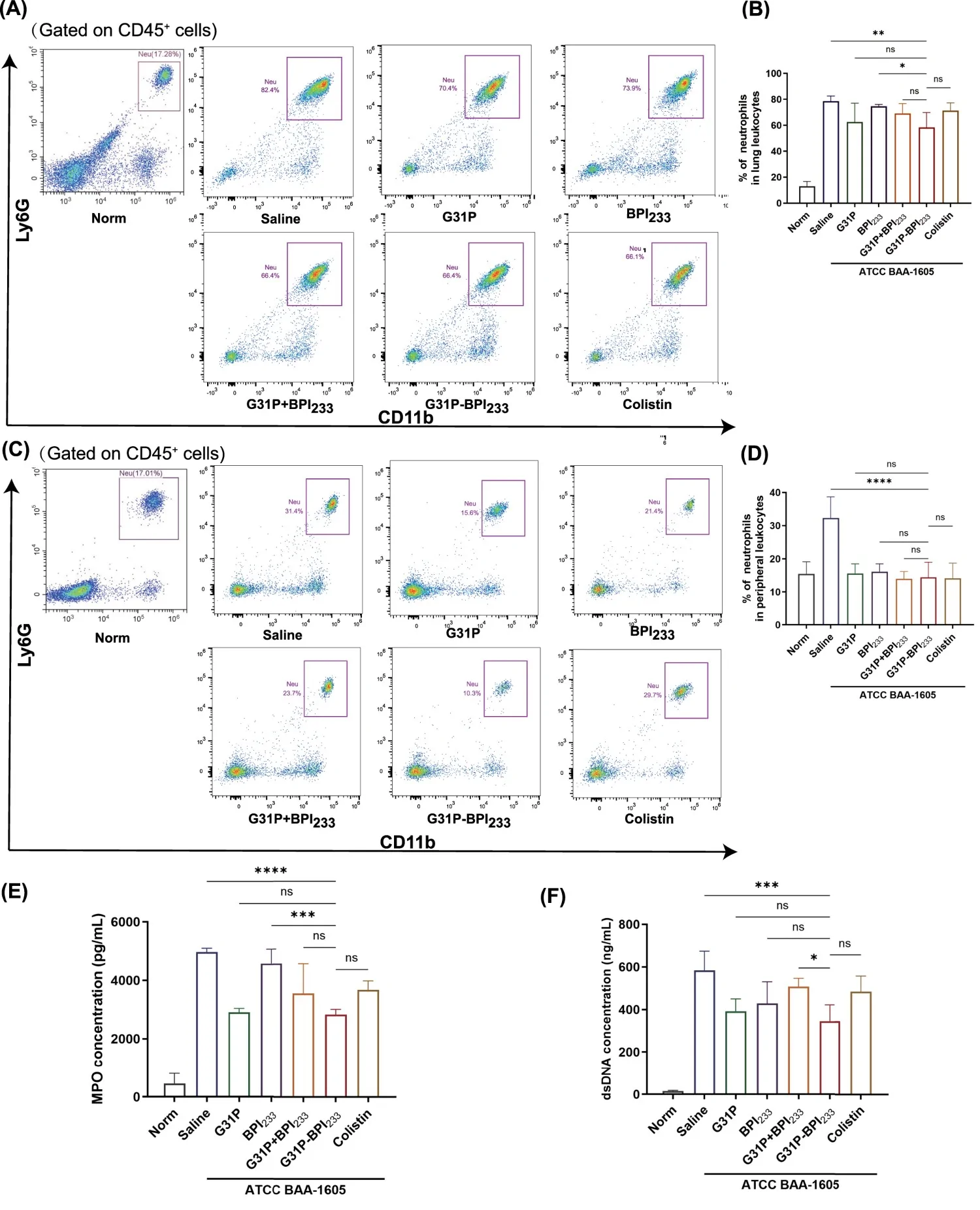
G31P–BPI_233_ limits neutrophil-associated inflammation during CRAB pneumonia. Mice were intranasally infected with *A. baumannii* ATCC BAA-1605 and treated with saline, G31P, BPI_233_, an equimolar G31P + BPI_233_ mixture, G31P–BPI_233_, or colistin (n = 5 per group). Uninfected mice served as normal controls (n = 3). Samples were collected 24 h after infection. (A–D) Representative flow-cytometry plots and quantification of CD45⁺CD11b⁺Ly6G⁺ neutrophils among lung (A, B) and peripheral blood (C, D) leukocytes. (E, F) Pulmonary levels of MPO and extracellular dsDNA. Component doses were molar-matched to the corresponding fusion-protein dose. Data are presented as mean ± SEM. ns, not significant; *P < 0.05; **P < 0.01; ***P < 0.001; ****P < 0.0001.

### G31p–BPI_233_ enhanced particle uptake by stimulated human neutrophils, increased apoptosis-like staining, and reduced NET-associated markers

To show how G31P–BPI_233_ affects neutrophil function, we tested particle uptake, apoptosis-like Annexin V/PI staining, and NET-related marker release in human neutrophils *in vitro*. Flow cytometry showed that G31P–BPI_233_ did not significantly alter basal neutrophil phagocytic activity under unstimulated conditions. Upon stimulation, however, G31P–BPI_233_ increased neutrophil bead uptake, with a significant enhancement observed in the bacteria-stimulated group (Figure 6A, B). We then measured neutrophil apoptosis using Annexin V/PI staining. G31P–BPI_233_ had no clear effect on spontaneous neutrophil apoptosis. However, after LPS or bacterial stimulation, G31P–BPI_233_ significantly increased the percentage of Annexin V/PI-positive neutrophils compared with saline treatment (Figure 6C, D), suggesting that G31P–BPI_233_ may help restore neutrophil apoptotic clearance under inflammatory conditions. In addition, NET-associated markers were measured by MPO and dsDNA release. PMA and bacterial stimulation clearly increased MPO and dsDNA levels. However, G31P–BPI_233_ treatment significantly reduced both markers (Figure 6E, F).

**Figure 6.**
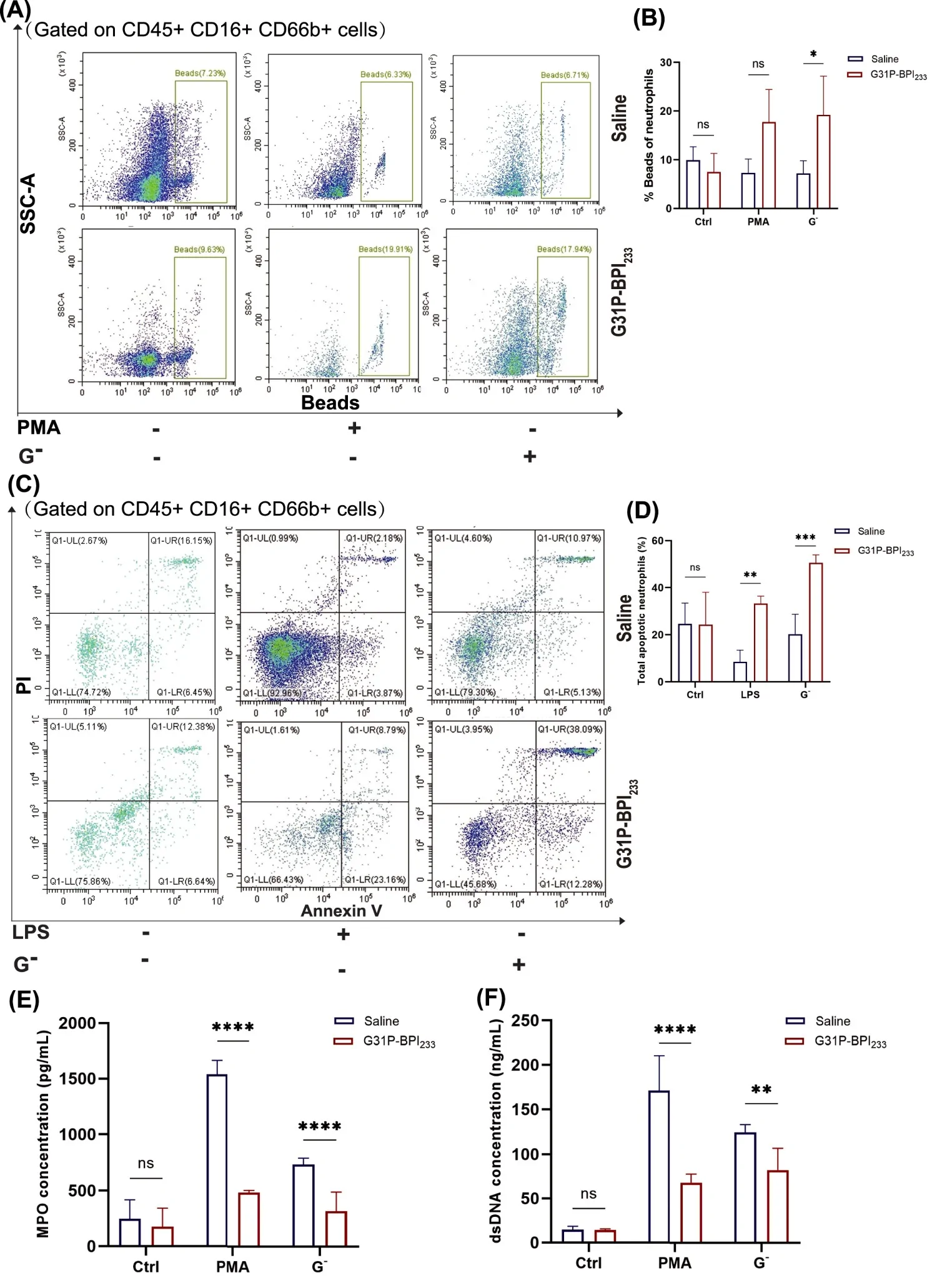
G31P–BPI_233_ enhances stimulated neutrophil particle uptake while limiting excessive activation and NET-associated marker release. (A, B) Percentages of bead uptake by CD45^+^ CD16^+^ CD66b ^+^ human neutrophils treated with saline or G31P–BPI_233_ under unstimulated conditions or after stimulation with PMA or bacteria. (C, D) Percentages of total apoptosis-like Annexin V/PI staining in human neutrophils treated with saline or G31P–BPI_233_ under control conditions or after stimulation with LPS or bacteria. (E, F) MPO (E) and extracellular dsDNA (F) levels released from human neutrophils treated with saline or G31P–BPI_233_ under control, PMA-stimulated, or bacteria-stimulated conditions. Data are presented as mean ± SEM. ns, not significant; *P < 0.05; **P < 0.01; ***P < 0.001; ****P < 0.0001.

### BPI deficiency increased bacterial burden and endotoxemia without altering neutrophil recruitment

To further study the role of endogenous BPI in bacterial killing and neutrophil regulation, WT and BPI^-/-^ mice were infected intranasally with CRAB. Tissues were collected at 1–3 dpi. Compared with WT mice, BPI^-/-^ mice had higher bacterial burdens in the lungs and spleen, with significantly increased levels at 1 and 2 dpi (Figure S8A–C). Plasma endotoxin levels were also significantly higher in BPI^-/-^ mice than in WT mice at 1 and 2 dpi (Figure S8D). However, flow cytometric analysis showed that the percentages of neutrophils in the lung and peripheral blood were comparable between WT and BPI^-/-^ mice during infection (Figure S8E-H). These results suggest that the impaired bacterial control observed in BPI^-/-^ mice was not primarily due to defective neutrophil recruitment.

### BPI deficiency disrupts neutrophil maturation but does not significantly affect neutrophil apoptosis *in vivo*

We then examined whether BPI deficiency affected neutrophil apoptosis and maturation *in vivo.* Annexin V/PI staining showed no significant difference in early or late apoptotic neutrophils between WT and BPI^-/-^ mice at 2 dpi. This suggests that BPI deficiency did not clearly change neutrophil apoptosis in the lung during the acute infection phase (Figure S9A–C). Analysis of BM neutrophil populations showed a clear defect in neutrophil maturation in BPI^-/-^ mice. Compared with WT mice, BPI^-/-^ mice had a higher percentage of preneutrophils and immature neutrophils, while a lower percentage of mature neutrophils (Figure S9D–G). These data indicate that BPI was involved in maintaining normal neutrophil maturation during bacterial infection.

### BPI deficiency impairs neutrophil particle uptake function and dysregulates neutrophil activation *in vitro*

To test whether BPI deficiency directly affects neutrophil function, BM-derived neutrophils from WT and BPI^-/-^ mice were isolated for *in vitro* assays. Compared with WT neutrophils, BPI^-/-^ neutrophils showed significantly reduced phagocytic capacity after stimulation, suggesting impaired antibacterial function (Figure S10A, B). Annexin V/PI staining also showed that, after stimulation, especially with bacteria, BPI^-/-^ neutrophils had higher percentages of both early and late apoptotic cells than WT neutrophils (Figure S10C–E). In addition, BPI^-/-^ neutrophils also showed stronger NET-related responses. After PMA or bacterial stimulation, dsDNA and MPO levels were higher in BPI-deficient neutrophils than in WT neutrophils, indicating increased NET-related marker release and neutrophil activation (Figure S10F, G).

## Discussion

Antibiotic resistance in bacteria is a growing concern worldwide. CRGNB, especially CRAB infections, are a serious threat worldwide that show no sign of abating due to limited therapeutic options[35]. Neutrophil-derived BPI is known for its bactericidal activity against Gram-negative bacteria and neutralization of lipopolysaccharide [36]. Given the relatively conserved nature of lipid A, BPI-mediated activity may extend to a broad range of Gram-negative bacteria irrespective of their antibiotic resistance profiles. In contrast, the likelihood of resistance development is expected to be relatively low. G31P is an engineered CXCL8 variant with antagonist activity toward CXCR1/CXCR2-mediated responses [37]. The role of CXCR1/2 as therapeutic targets has been underscored in previous studies, and G31P has been proven to be efficacious in many inflammatory diseases, including uric acid nephropathy, bacterial pneumonia, ulcerative colitis, and aspiration pneumonia [25,37–39].

In this study, we successfully generated G31P–BPI_233_ and performed preliminary characterization of its molecular, structural, pharmacokinetic, biochemical, and safety properties. Western blot identified a predominant band at the expected molecular weight. AlphaFold2/ColabFold predicted a two-domain architecture connected by a flexible linker. The fusion protein remained detectable in the circulation, with an apparent half-life of approximately 1.3 h, and a band corresponding to the expected fusion-protein size remained detectable after incubation in 90% mouse serum at 37°C for up to 24 h. Moreover, administration at the therapeutic dose produced no detectable acute systemic toxicity or liver or kidney injury during the 24-h observation period. Together, these findings support the expected molecular size, serum persistence, and preliminary short-term tolerability of G31P–BPI_233_ under the tested conditions (Figure 7).

**Figure 7.**
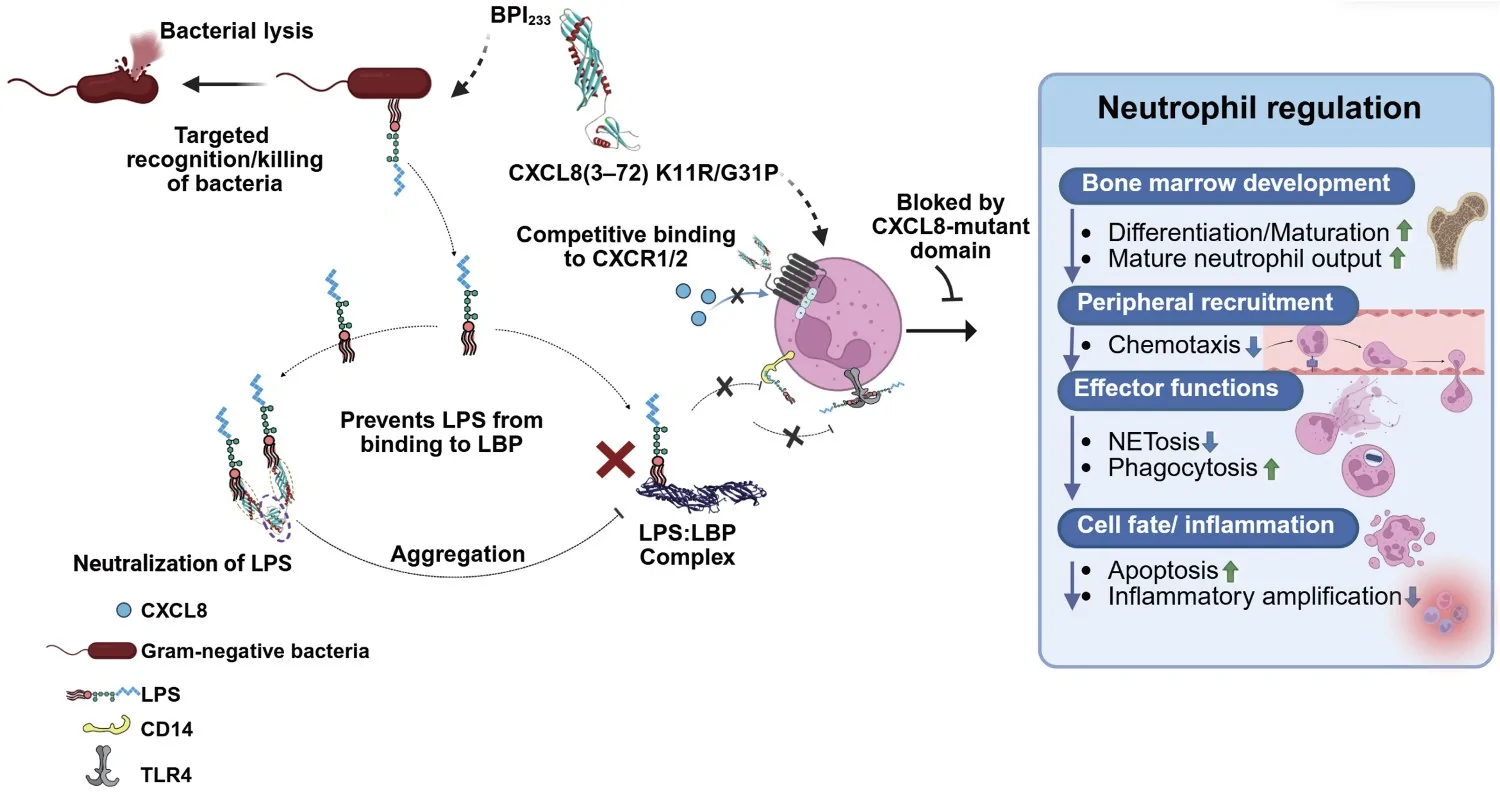
**Proposed mechanism by which G31P–BPI233 protects against Gram-negative bacteria infection.** G31P–BPI_233_ combines BPI_233_-mediated bacterial killing and LPS neutralization with G31P-mediated inhibition of CXCL8–CXCR1/2-driven neutrophil chemotaxis. This dual activity reduces bacterial/endotoxin burden and limits excessive neutrophil-associated inflammation while preserving antibacterial effector functions.

An important consideration in the development of multifunctional fusion proteins is whether covalent linkage preserves the biological activities of the constituent domains [40]. G31P–BPI_233_ retained the principal functions of both components, including BPI_233_-associated endotoxin neutralization and broad-spectrum Gram-negative bactericidal activity, together with G31P-associated inhibition of CXCL8-induced human neutrophil migration. It also reduced LPS-induced IL-6 and TNF-α production in PBMCs. Similar functional integration has been reported for BPI21/LL-37-based constructs and other multifunctional anti-infective fusion biologics, in which preservation of constituent activities together with therapeutic efficacy in infection models supported the rationale for fusion-based design [16,26,41]. Thus, the present findings support G31P–BPI_233_ as a functionally integrated bifunctional molecule rather than implying that covalent fusion necessarily generates pharmacological synergy in each individual *in vitro* assay.

Consistent with this concept, the component-equivalent comparisons primarily demonstrated functional preservation rather than uniform enhancement of potency after fusion. Direct bactericidal and LPS-responsive effects were largely associated with the BPI_233_-containing preparations, whereas inhibition of CXCL8-induced chemotaxis was associated with the G31P-containing preparations. The comparable activity of G31P–BPI_233_ and the noncovalent mixture in several in vitro assays therefore indicates that covalent fusion preserved, rather than uniformly enhanced, the principal activities of the constituent domains. Importantly, the therapeutic value of a multifunctional fusion protein may become more apparent *in vivo*, where pathogen burden, endotoxin exposure, inflammatory signalling, pharmacokinetics, and host-cell responses occur simultaneously.

Indeed, this distinction became more apparent in the CRAB pneumonia model. G31P–BPI_233_ simultaneously reduced bacterial burden, residual endotoxin activity, inflammatory cytokines, and lung injury. Although the between-group differences in bacterial burden and endotoxin activity did not reach statistical significance, both endpoints were numerically lower with G31P–BPI233 than with the equimolar noncovalent mixture. Moreover, G31P–BPI_233_ produced significantly greater reductions in pulmonary IL-6 and TNF-α than BPI_233_ alone or the mixture. These findings suggest an endpoint-specific in vivo advantage of covalent fusion, with a favourable numerical pattern across antibacterial and endotoxin-related outcomes and a statistically significant advantage in selected inflammatory endpoints. Covalent linkage may facilitate fixed-stoichiometric co-delivery and may also influence stability, tissue exposure, biodistribution, or pharmacokinetic behaviour; however, direct comparative pharmacokinetic studies of the fusion and noncovalent preparations will be required to determine the underlying mechanism.

Neutrophil regulation may be an important part of the protective effect of G31P–BPI_233_. During CRAB pneumonia, excessive neutrophil activation can cause lung injury by releasing proteases, oxidants, and NET-related components. In this study, G31P–BPI_233_ reduced pulmonary MPO and extracellular dsDNA levels. This suggests that G31P–BPI_233_ reduced excessive neutrophil activation and NET-related inflammatory responses. In human neutrophils, G31P–BPI_233_ increased bead uptake after bacterial stimulation. It also reduced MPO and dsDNA release. This suggests that G31P–BPI_233_ may preserve or enhance antibacterial activity and reduce neutrophil responses that damage tissues. The seemingly paradoxical combination of reduced neutrophil accumulation, decreased extracellular MPO and dsDNA, yet lower bacterial burden warrants integrated interpretation. First, the reduction in circulating and pulmonary neutrophils is consistent with G31P-mediated blockade of CXCL8–CXCR1/2-driven chemotaxis, which limits excessive neutrophil influx without abrogating baseline recruitment. Second, decreased MPO and dsDNA levels in lung homogenates reflect attenuated neutrophil degranulation and NETosis, rather than impaired intracellular killing. Third, the G31P–BPI_233_-treated neutrophils that do infiltrate the lung exhibit enhanced phagocytic capacity, suggesting a functional shift from a degranulation/NETosis-prone phenotype toward a phagocytic bactericidal phenotype. Fourth, the BPI_233_ domain provides direct bactericidal activity and endotoxin neutralization independently of host effector function. Finally, reduced extracellular MPO does not preclude intraphagosomal killing, which is sustained by NADPH oxidase-derived ROS and non-oxidative effectors such as defensins and lysozyme. Collectively, these data suggest that G31P–BPI_233_ modestly limits neutrophil-associated inflammation by favouring phagocytic function over neutrophil influx and degranulation in the host response.

The BPI^-/-^ mouse experiments provided more mechanistic information. BPI deficiency increased bacterial burden at the main infection site. It also promoted later systemic spread and increased plasma endotoxin levels. These results support an important role of endogenous BPI in controlling Gram-negative bacterial infection and preventing endotoxemia. Interestingly, BPI^-/-^ mice did not show a clear defect in neutrophil recruitment to the lung or blood. This suggests that poor bacterial control was not primarily due to impaired neutrophil motility. Instead, BPI deficiency was associated with abnormal neutrophil maturation in the BM, reduced phagocytic capacity, and increased release of NET-related markers.

However, several limitations should be noted. First, although longer than rBPI23 (8.2 min) [42], the serum half-life of G31P–BPI_233_ was relatively short (1.3 h). This pharmacokinetic profile may restrict direct clinical translation if frequent systemic dosing is required [16]. Therefore, future engineering optimization will be necessary to prolong the circulation time of G31P–BPI_233_. Several established half-life extension strategies could be considered, including PEGylation, Fc fusion, albumin fusion, fusion to an albumin-binding domain, or genetically encoded hydrophilic polypeptide extensions [43]. In addition, nanoparticle, liposomal, or local pulmonary delivery may further improve pulmonary exposure while reducing the need for frequent systemic dosing. Second, the present study evaluated one selected domain orientation and linker design. It was designed to establish proof of concept for functional integration rather than to exhaustively optimize fusion architecture. The preservation of both constituent activities supports the feasibility of this construct, but comparisons of alternative linkers, domain orientations, or stability-enhancing modifications may identify improved variants in future engineering studies [40]. Finally, mice lack a direct CXCL8/IL-8 ortholog and primarily express the CXCR2 receptor (39). Therefore, the *in vivo* effects of G31P–BPI_233_ in BALB/c mice cannot directly prove CXCL8–CXCR1/CXCR2 blockade. Future studies on protection and mechanisms using guinea pig pneumonia models may help better define the role of the G31P-mediated ELR-CXC chemokine axis.

In conclusion, G31P–BPI_233_ represents a bifunctional anti-infective fusion protein that preserves BPI_233_-associated Gram-negative bacterial killing and endotoxin neutralization while retaining G31P-associated inhibition of CXCL8-driven neutrophil chemotaxis. Rather than producing uniform enhancement across individual *in vitro* assays, the fusion integrates these complementary activities within a single molecule and provides coordinated antibacterial and inflammation-modulating effects *in vivo*. By simultaneously reducing pathogen burden and limiting neutrophil-associated inflammatory injury, G31P–BPI_233_ represents a promising host-directed antibacterial strategy for severe CRAB pneumonia.

## Supplementary Material

Figure S3_0910.eps

Figure S8_0910.eps

Figure S4_0910.eps

Table S2.docx

Figure S9_0910.eps

Figure S2_0910.png

Table S1.docx

Figure S5_0910.eps

Figure S6_0910.eps

Figure S1_0910.eps

Figure S7_0910.eps

## Data Availability

The data that support the findings of this study are available from the corresponding author upon reasonable request.
