# Supplementary material for: A novel CXCL8(3–72) K11R/G31P–BPI233 hybrid protein modulates neutrophil-driven inflammation and protects against carbapenem-resistant Acinetobacter baumannii infection
Source: Emerg Microbes Infect. 2026 Oct 15;15(1):2741787. doi: 10.1080/22221751.2026.2741787 (PMC13674666; doi:10.1080/22221751.2026.2741787)
Supplement: Supplementary file 1 [file TEMI_A_2741787_SM7017.docx]

**Table S1.** Clinical and antimicrobial susceptibility characteristics of the five clinical CRAB isolates used in this study.

| Isolates No. | Isolation date | | Hospital/Region^a^ | | | Ward | | Specimen  source | Clinical context | | **AST results^b^** | | | | | | | | | | | | | | | | | | | | | |  | | |
| --- | --- | --- | --- | --- | --- | --- | --- | --- | --- | --- | --- | --- | --- | --- | --- | --- | --- | --- | --- | --- | --- | --- | --- | --- | --- | --- | --- | --- | --- | --- | --- | --- | --- | --- | --- |
|  |  |  |  |  |  |  |  |  |  |  | **SAM** | **AMK** | **TGC** | | **TIM** | **CSL** | **TZP** | **CAZ** | **FEP** | **IPM** | | | **MEM** | **CIP** | **LEV** | **TOB** | **SXT** | | **MIN** | | **CST** | |  |  |  |
| 99389 | 2026/07/09 | | CJFH, Beijing | | MICU | | BALF | | Pneumonia | | **>=256** | **6^c^** | 17^c^ | | **>=128** | **>=64** | **>=128** | **>=64** | **>=32** | **>=16** | | | **>=16** | **>=4** | **>=8** | **>=16** | **>=320** | | **8** | | <=0.5 | |  |  |  |
| 99391 | 2026/07/09 | | CJFH, Beijing | | MICU | | BALF | | Pneumonia | | **>=256** | **6^c^** | 2 | | **>=128** | **>=64** | **>=128** | **>=64** | **>=32** | **>=16** | | | **>=16** | **>=4** | **>=8** | **>=16** | **>=320** | | **8** | | <=0.5 | |  |  |  |
| 99392 | 2026/07/09 | | CJFH, Beijing | | MICU | | BALF | | Pneumonia | | **>=256** | **6^c^** | 1 | | **>=128** | 32 | **>=128** | **>=64** | **>=32** | **>=16** | | | **>=16** | **>=4** | 4 | **>=16** | **>=320** | | **4** | | <=0.5 | |  |  |  |
| 99393 | 2026/07/09 | | CJFH, Beijing | | MICU | | BALF | | Pneumonia | | **>=256** | **6^c^** | <=0.5 | | **>=128** | 32 | **>=128** | **>=64** | **>=32** | **>=16** | | | **>=16** | **>=4** | 4 | **>=16** | <=20 | | **8** | | 2 | |  |  |  |
| 99394 | 2026/07/09 | | CJFH, Beijing | | MICU | | Sputum | | Pneumonia | | **>=256** | **6^c^** | 2 | | **>=128** | 32 | **>=128** | **>=64** | **>=32** | **>=16** | | | **>=16** | **>=4** | 4 | **>=16** | **>=320** | | **4** | | <=0.5 | |  |  |  |
|  |  |  | |  | | | | | |  | | | | | | | | | | | |  |  |  |  |  |  |  |  |  |  |  |  |  |  |
|  |  | |  | | |  |  | |  | |  |  | |  |  |  |  |  |  | |  | |  |  |  |  | |  | |  | |  | |  |  |

^a^ CJFH, China-Japan Friendship Hospital; ^b^ SAM, ampicillin/sulbactam; AMK, amikacin; TGC, tigecycline; TIM, ticarcillin/clavulanate; CSL, cefoperazone/sulbactam; TZP, piperacillin/tazobactam; CAZ, ceftazidime; FEP, cefepime; IPM, imipenem; MEM, meropenem; CIP, ciprofloxacin; LEV, levofloxacin; TOB, tobramycin; SXT, trimethoprim/sulfamethoxazole; MIN, minocycline; CST, colistin. Unless otherwise indicated, antimicrobial susceptibility values are minimum inhibitory concentrations (MICs, μg/mL). ^c^ Values indicate a disk diffusion zone diameter (mm). Bold values were reported as resistant (R);
