# Supplementary material for: A novel CXCL8(3–72) K11R/G31P–BPI233 hybrid protein modulates neutrophil-driven inflammation and protects against carbapenem-resistant Acinetobacter baumannii infection
Source: Emerg Microbes Infect. 2026 Oct 15;15(1):2741787. doi: 10.1080/22221751.2026.2741787 (PMC13674666; doi:10.1080/22221751.2026.2741787)
Supplement: Supplementary file 2 [file TEMI_A_2741787_SM7020.docx]

**Table S2.** Fusion protein CXCL8_(3-72) K11R/G31P_-BPI_233_ sequence

| **Component** | **Sequence** |
| --- | --- |
| CXCL8_(3-72) K11R/G31P_ | KELRCQCIRTYSKPFHPKFIKELRVIESPPHCANTEIIVKLSDGRELCLDPKENWVQRVVEKFLKRAENS |
| Linker | GGGSGGGSGGGS |
| BPI_233_ | VNPGVVVRISQKGLDYASQQGTAALQKELKRIKIPDYSDSFKIKHLGKGHYSFYSMDIREFQLPSSQISMVPNVGLKFSISNANIKISGKWKAQKRFLKMSGNFDLSIEGMSISADLKLGSNPTSGKPTITASSCSSHINSVHVHISKSKVGWLIQLFHKKIESALRNKMNSQVCEKVTNSVSSELQPYFQTLPVMTKIDSVAGINYGLVAPPATTAETLDVQMKGEFYSENH |
